# Characteristics, outcomes, and maternity care experiences of women with children’s social care involvement who subsequently died: national cohort study and confidential enquiry

**DOI:** 10.1136/bmjmed-2025-001464

**Published:** 2025-07-10

**Authors:** Kaat De Backer, Allison Marjorie Felker, Emma Rose, Caroline Bull, Oluwaseun Labisi, Kirsty Kitchen, Claire Mason, Elsa Montgomery, Jane Sandall, Abigail Easter, Marian Knight, Nicola Vousden

**Affiliations:** 1 Department of Women and Children’s Health, Faculty of Life Sciences and Medicine, King’s College London, London, UK; 2 National Perinatal Epidemiology Unit, University of Oxford, Oxford, UK; 3 Rosie Hospital, Cambridge University Hospitals NHS Foundation Trust, Cambridge, UK; 4 Royal College of Midwives, London, UK; 5 Croydon University Hospital, London, UK; 6 Birth Companions, London, UK; 7 Centre for Child and Family Justice Research, Lancaster University, Lancaster, UK; 8 Methodologies Division, Florence Nightingale Faculty of Nursing, Midwifery and Palliative Care, King’s College London, London, UK

**Keywords:** Child protective services, Health services, Obstetrics, Prenatal care, Quality of health care

## Abstract

**Objectives:**

To investigate maternal mortality in the context of children's social care (CSC) involvement, and to explore the quality of maternity care that women with CSC involvement received.

**Design:**

National cohort study and confidential enquiry.

**Setting:**

MBRRACE-UK (Mothers and Babies: Reducing Risk through Audits and Confidential Enquiries across the UK) national surveillance dataset for deaths that occurred during pregnancy or up to a year after pregnancy, UK, 2014-22.

**Participants:**

1451 women who died during or in the year after pregnancy in the UK; 420 women (28.9%) had CSC involvement. 47 women's healthcare records were included in the confidential enquiry to describe the care of a random sample of women who died during the perinatal period who had CSC involvement.

**Main outcome measures:**

Rates and causes of maternal deaths by CSC involvement and quality of care.

**Results:**

A third (420/1451, 28.9%) of the women who died during or in the year after pregnancy had CSC involvement for their (unborn) baby. Women with CSC involvement were more likely to be aged ≤20 years (rate ratio 1.85, 95% confidence interval 1.27 to 2.63, compared with those aged 21-29 years), living in the most deprived areas (rate ratio 2.19, 1.42 to 3.50, compared with those least deprived), and less likely to be from black (rate ratio 0.56, 0.35 to 0.84) or Asian ethnic backgrounds (rate ratio 0.26, 0.14 to 0.44, compared with white women) than women who died with no known CSC involvement. Deaths occurred predominantly between six weeks and the year after pregnancy (75%), and higher proportions of deaths were caused by suicide, other psychiatric causes, including substance overdose, and homicide. A confidential enquiry identified that risk assessment and recognition, medication management, coordination of care, and staff competencies were essential components in providing personalised, holistic, and trauma-informed care when dealing with medical and social complexity. Multiple individual and systemic barriers hindered access and engagement with healthcare.

**Conclusions:**

Women with CSC involvement who died during or in the year after pregnancy encountered multiple inequalities and were at an increased risk of maternal mortality from psychiatric causes and homicide. A critical review of current care pathways and policy changes is urgently needed to tailor care to the needs of this group of women and to look at the inequalities that disproportionately affect them.

**WHAT IS ALREADY KNOWN ON THIS TOPIC**

- Involvement of children's social care (CSC) during pregnancy and among infants aged <1 year has increased in the past decade
- Previous confidential enquiries into maternal deaths in the UK have described how women with multiple adversity encounter many biases, affecting the quality of maternity care that they receive
- A proportion of these women will also have CSC involvement, known to be associated with increased maternal mortality and morbidity and, for some, removal of their infant from their care

**WHAT THIS STUDY ADDS**

- Differences in sociodemographic and clinical characteristics, time of death, and causes of death between women with CSC involvement and those without were found, based on UK maternal mortality data, 2014-22
- Essential components to provision of care, ensuring personalised, holistic, and trauma informed care, were identified
- Uncoordinated appointment schedules across a wide number of services became an additional challenge for women who already faced many disadvantages

**HOW THIS STUDY MIGHT AFFECT RESEARCH, PRACTICE, OR POLICY**

- These findings can be used to inform changes to policy and practice to improve care for this group of marginalised women
- A critical review of current maternity care pathways is needed to adjust and customise care to the needs of women with complex social adversity, and to look at the existing health inequalities that disproportionately affect this group of women

## Introduction

 In many countries, safeguarding legislation and processes are in place to ensure children's safety and wellbeing. In the UK, children's social care (CSC) might become involved during pregnancy or after birth when safeguarding concerns are raised that a baby might be at significant risk of harm after birth.^1^ The latest MBRRACE-UK (Mothers and Babies: Reducing Risk through Audits and Confidential Enquiries across the UK) report, including national surveillance data of maternal deaths in the UK from 2020 to 2022, reported that 22% of women who died during pregnancy or in the six weeks after the end of their pregnancy were known to CSC.^2^ This finding is nearly a twofold increase from 12% in the 2012-14 triennium.^3^ A similar trend of rising rates of CSC involvement during pregnancy and among infants aged <1 year can be found in England through the Child in Need census data from the Office for National Statistics.^4^ Recent work based on the Child in Need census data estimated that by age 18 years, one in four children in England are identified by CSC as needing support at some point.^5^

Although maternal deaths are uncommon in the UK (12.67 per 100 000 maternities, 95% confidence interval 11.00 to 14.33, in 2021-23), recent evidence indicates a substantial increase compared with earlier periods, even if deaths from covid-19 disease are excluded.^2^ Inequalities in maternal mortality have been consistently reported for black and Asian women, as well as for those from the most deprived areas. Women facing multiple adversity are also over-represented among women who die.^2 6^ Confidential Enquiries into maternal deaths seek to identify systemic changes to improve care, and not to apportion individual blame, and the 2020 MBRRACE-UK Confidential Enquiry report noted that 90% of women who died had a "constellation of biases", as a result of physical comorbidities, mental health problems, and a range of complex social risk factors, such as deprivation, contact with the criminal justice system, or domestic abuse by a partner or ex-partner.^7^ This observation is particularly relevant for women with CSC involvement, because referrals are often made as a result of concerns about various (co-existing) social risk factors. Of note in this paper, we use the term domestic abuse in line with the statutory definition of domestic abuse in the UK Domestic Abuse Act, 2021. This term includes any incident or pattern of incidents of controlling, coercive, or threatening behaviour. In contrast with the term domestic violence or inter-partner violence, domestic abuse is broader and takes many forms, including emotional, physical, economic, and financial abuse or coercive control.

CSC involvement is associated with increased maternal morbidity^8^ and mortality.^9 10^ Evidence from Canada and Sweden indicated that women who had an infant compulsory removed from their care because of safeguarding concerns were three times more likely to die from both avoidable and unavoidable causes.^9 10^ Evidence is limited, however, for describing the characteristics and outcomes of this cohort. Our study used national maternal mortality surveillance data and confidential enquiry methodology to describe the characteristics and care received by women in the UK with CSC involvement who subsequently died. This study had two objectives: to describe the sociodemographic, clinical, social, and pregnancy-related characteristics and causes of death of women with CSC involvement who died in the UK during or up to a year after the end of pregnancy between 2014 and 2022; and to conduct a confidential enquiry of anonymised medical records of a sample of women who died and who had CSC involvement, to explore the quality of maternity care they received.

## Methods

### Women who died

The MBBRACE-UK collaboration is responsible for delivering the Maternal, Newborn, and Infant Clinical Outcome Review Programme (MNICORP) that conducts enhanced surveillance of all maternal deaths in the UK during or up to one year after pregnancy. Data held by MBRRACE-UK are crosschecked against national death and birth registries to ensure accuracy and completeness, and triennial maternal death reviews are published in accordance with the World Health Organization Maternal Death Surveillance and Response guidance.^11^ Maternal deaths are predominantly reported to MBRRACE-UK by the hospital where the mother died, or alternatively by coroners, pathologists, or members of the public, cross checked with linked birth and death vital statistics. For each woman who dies, MBRRACE-UK collects information and documentation, including: a surveillance form, completed by a professional in maternity services involved in the woman's care; maternity antenatal notes and hospital records; and any other healthcare records, such as general practice records, coroners' reports, post-mortem reports, and mental health and social care records. Information identifying the woman, their family, professionals, and services involved is redacted to ensure confidentiality and anonymity.

For this project, the MBRRACE-UK dataset was used to sample all deaths that occurred during pregnancy or up to a year after pregnancy in the UK from 2014 to 2022. Permission to use the MBRRACE-UK dataset for this analysis was provided by the Healthcare Quality Improvement Partnership (HQIP), after approval of an extended analysis and output request. Two questions from the MBRRACE-UK surveillance form were used to identify the cohort with CSC involvement: was the woman known to CSC and was the newborn infant taken or to be taken into care? If this information was completed on the surveillance form, these women were included in the analysis; if this information was missing, the women were excluded from our analysis.

For the purpose of this analysis, we defined CSC involvement as active or ongoing involvement of CSC, during pregnancy or the postnatal period, for the unborn or newborn child, before the woman's death. The involvement might have been extended to older children in the family. According to relevant sections of the Children’s Act 1989, involvement might have been about voluntary offers of support (s.17), mandatory child protection (s.47), court ordered removals (s.31), or voluntary arrangements between CSC and the child's parents (s.20).^1^

Data for personal, social, medical, and pregnancy-related characteristics of all women eligible for inclusion were extracted from the MBRRACE-UK national surveillance database.^2 12^ Routine surveillance data captured by MBRRACE-UK were cleaned and, in some instances, re-categorised into binary or aggregate groups to conduct direct comparisons. Data for ethnic groups were grouped as five aggregated categories used in the UK census classification (Asian, black, mixed or multiple ethnic group, other, or white) because of small numbers in some of the more detailed ethnic group categories among women with CSC involvement.^13^ Medical and obstetric risk factors were coded according to ICD (international classification of diseases) codes if known. A delay in starting antenatal care was defined according to the NHS key performance indicator for antenatal assessments, which should occur before 13 weeks' gestation.^14^ Causes of death were classified according to MBRRACE-UK reporting and ICD maternal mortality subgroups.^15^ Socioeconomic status was derived from the index for multiple deprivation (divided into five groups), with group I being the most deprived and group V being the least deprived.^16^

### Confidential enquiry cohort

**Figure 1:**
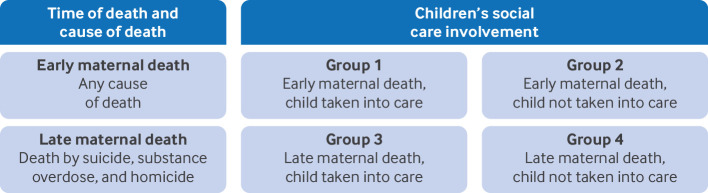
Sampling for confidential enquiry into maternal deaths with children’s social services involvement

To investigate the quality of care of women who died with CSC involvement, a confidential care review of anonymised case notes was conducted specifically, as recommended in WHO Maternal Death Surveillance and Response (MDSR) guidance^17^ and in line with previous work exploring the care of underserved groups of women. ^18^ Previous MBRRACE-UK reports have consistently reported that suicide and substance overdose were the leading causes of death among women who died between six weeks and one year after the end of pregnancy, and a disproportionately high number of these women had CSC involvement.^19^ To ensure that this confidential enquiry reviewed a range of CSC involvement, four subgroups were created based on the level of involvement (ie, whether or not the infant went into care, by court order or parental agreement), time of death, cause of death, and with geographical representation across England, Scotland and Wales, in keeping with known maternal mortality rates (figure 1). For each subgroup, 20 sets of case notes were randomly sampled for an in-depth case note review. At the time of sampling for the confidential enquiry, the individual case records for maternal deaths that occurred in 2022 were not available, therefore samples were taken from women who died in 2014 to 2021. At the time these data became available, reviewers had reached a consensus about the key findings and it was decided that no further sampling was required.

The terms "pregnant women" and "mothers" will be used throughout this paper to reflect the recorded characteristics of individuals identified in the MBRRACE-UK case notes, but the authors recognise not everyone who is pregnant or giving birth will identify as a woman or a mother.

### Statistical methods

For the first objective, descriptive statistical methods were used to investigate sociodemographic, clinical, social, and pregnancy-related characteristics and causes of death. The incidence of each variable, including specific causes of death, is presented (number and percentage) for each of the two groups of interest (women with known CSC involvement and women with no known CSC involvement). For each variable, a χ^2^ test was used to compare the proportions between the two groups. Sociodemographic covariates to identify different population groups included maternal age, social deprivation, and ethnic group.

Incidence rates per 100 deaths and 95% confidence intervals (CIs) for the proportion of women with CSC involvement who belonged to specific population groups (age, index of multiple deprivation group, and ethnic group) were calculated, with the total number of deaths in each group as the denominator (excluding deaths where CSC involvement was not known). Baseline groups were determined based on standard MBRRACE-UK reporting.^2^ With the incidence rate ratio calculator command in Stata (iri), the incident rates by CSC involvement for different population groups (age, index of multiple deprivation group, and ethnic group) among women who died were calculated. All data analyses were conducted with Stata 17. Significance was set at a P value of <0.05.

### Confidential enquiry methodology

For the second objective, a confidential enquiry was carried out. This methodology uses a combination of qualitative and quantitative analysis methods, taking account of medical and non-medical factors that led to a woman’s death. The strength of confidential enquiry methodology lies in the aggregation of data on individual cases to show common factors across a particular cohort, for which remedial action may be possible.^20^ To this purpose, a data extraction template was co-designed with input from a multidisciplinary steering group and lived experience panel, to facilitate extraction of information based on personal and clinical characteristics, complex social risk factors, and CSC involvement. Online supplemental table S1 outlines how information about complex social risk factors was extracted into categories, according to a previous MBRRACE-UK case note review on social risk factors.^21^ The template also had key areas of good care for women with CSC involvement, to facilitate thematic analysis of quality of maternity care. These themes were derived and merged from two recent documents, co-produced with women with lived experience (ie, birth charter for women with involvement from children's social care^22^ and the Born Into Care: best practice guidelines for when the state intervenes at birth).^23^ Both documents outline a series of standards and principles to improve care for women with CSC involvement, developed through extensive input of people with lived experience. A list of prompts accompanied the template to ensure assessors had a similar approach to data collection and extraction. These are shown in online supplemental table S2.

Relevant information on the items included in the extraction template was entered into an Excel spreadsheet. All anonymised case notes were reviewed by one assessor (first author), and two thirds of the case notes were reviewed by a second member of the assessor's team. In accordance with WHO MDSR guidance, the panel was made up of multidisciplinary expertise to assess medical and non-medical factors influencing care.^17^ This expertise consisted of clinical academics and clinicians with a background in obstetrics, midwifery, perinatal mental health, domestic abuse and substance misuse, safeguarding, care quality and standards, and public health. Relevant information for each key theme was summarised in narrative form by every assessor. Assessors discussed each case collectively, identifying minimal differences in <5% of extracted fields and agreeing on key findings by consensus. Findings were thematically grouped, and themes were iteratively discussed with the wider supervisory team, steering group, as well as with women with lived experience, to facilitate sense checking and prioritisation of key themes and sub-themes.

### Patient and public involvement

This work is part of a wider doctoral research study on maternity care of women with CSC involvement (MUMS@RISC study). The MUMS@RISC advisory panel of six women with lived experience of infant removal contributed to study design, with one of them also representing the lived experience voice in the steering group. With support from Birth Companions and the Born Into Care research group, preliminary findings were presented and discussed with a wider group of women with lived experience of infant removal, either through individual discussions or in a group based format. At all times, trauma-informed principles of research engagement^24^ were respected, to create a safe environment to reflect on and discuss the care that women had received. Feedback from patient and public involvement informed the visual display, providing an overview of key themes.

## Results

### Cohort characteristics

**Figure 2:**
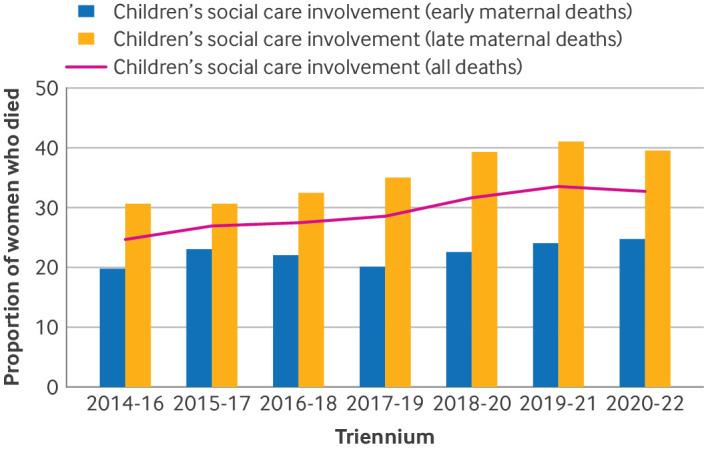
Proportion of women who died with children's social care (CSC) involvement. Proportions of women who died with CSC involvement in each overlapping triennia do not include women who had missing data. Observed increase in proportion is independent of missing values

Of the 1695 maternal deaths between 2014 and 2022, CSC involvement status was not known for 244 women. Therefore, our final sample was 1451 women who died during or in the year after pregnancy in the UK, 2014-22. Within this sample, 420 women (28.9%) had CSC involvement. Figure 2 shows the proportion of women who died with and without CSC involvement, for each overlapping triennium. The proportion of CSC involvement among maternal deaths has been steadily increasing since 2014, with the highest proportion (33.7%) in women who died in 2019-21.

Women known to have CSC involvement were more likely to be unemployed (61% *v* 10%, employment status for both the woman and woman's partner), single (ie, with no partner, 23% *v* 7%), and living alone (32% *v* 6%) (table 1) than women with no known CSC involvement. Of those with CSC involvement, 65% (n=205) reported domestic abuse before or during pregnancy, compared with 3% (n=23) of women with no known CSC involvement. Similarly, the prevalence of disclosure of abuse during childhood was 33% (n=49) for women with CSC involvement compared with 2% (n=11) among those with no known involvement, although this information was missing for a large proportion of women in both groups (64.5% and 52.7% respectively). Online supplemental tables S3 and S4 describe the prevalence of missingness for variables within each group.

**Table 1:** Sociodemographic characteristics of women who died, 2014-22, UK*

Characteristics (n=1451 women who died)*	No known CSC involvement (n=1031)	Known CSC involvement (n=420)	P value between groups
Partner:			
Yes	953 (93)	316 (77)	<0.001
No	71 (7)	94 (23)	—
Missing	7	10	—
Socioeconomic status (occupational classification):			
Employed (woman or partner)	825 (90)	125 (39)	<0.001
Unemployed (both woman and partner)	88 (10)	198 (61)	—
Missing	118	97	—
Living arrangements:			
With partner, parents, or extended family	930 (94)	235 (68)	<0.001
Living alone	63 (6)	109 (32)	—
Missing/not known	38	76	—
Domestic abuse (before or during pregnancy):			
Yes	23 (3)	205 (65)	<0.001
No	737 (97)	109 (35)	—
Missing	272	106	—
History of abuse as a child:			
Yes	11 (2)	49 (33)	<0.001
No	477 (98)	100 (67)	—
Missing	543	271	—

Values are number (%). Calculated percentages do not include missing values.

P values are no known children's social care involvement group versus known children's social care involvement group.

* Does not include women whose involvement with children's social care was not known (n=244).

CSC, children's social care.

When reviewing rates of CSC involvement among different population groups (table 2), women aged ≤20 years were almost twice as likely as women from older age groups to have CSC involvement (rate ratio 1.85, 95% CI 1.27 to 2.63). Women from the most deprived areas were similarly twice as likely as women living in the least deprived areas (rate ratio 2.19, 95% CI 1.42 to 3.50) to have CSC involvement. Women from ethnic backgrounds other than white were less likely to have CSC involvement than their white counterparts, although low numbers of women from mixed or multiple ethnic backgrounds might have introduced variation in the findings.

**Table 2:** Rates of children's social care involvement among different groups of women who died, 2014-22, UK*

Characteristics	Total deaths, UK 2014-22*	No known CSC involvement (n=1031) (No (%))†	Known CSC involvement (n=420) (No (%))†	Known CSC involvement (rate per 100 deaths (95% CI))	Rate ratio (95% CI)	P value
Age (years):						
≤20	70	31 (3)	39 (9)	55.71 (43.34 to 67.59)	1.85 (1.27 to 2.63)	0.001
21-29	544	380 (37)	164 (39)	30.15 (26.32 to 34.20)	1 (reference)	—
30-39	690	502 (49)	188 (45)	27.25 (23.10 to 30.73)	0.90 (0.73 to 1.12)	0.344
≥40	147	118 (11)	29 (7)	17.73 (13.63 to 27.09)	0.65 (0.42 to 0.98)	0.029
Social deprivation (index of multiple deprivation group):†						
I (most deprived/lowest 20%)	473	293 (32)	180 (50)	35.05 (33.66 to 42.60)	2.19 (1.42 to 3.50)	<0.001
II	300	212 (23)	88 (25)	29.33 (24.24 to 34.84)	1.69 (1.06 to 2.77)	0.019
III	221	182 (20)	39 (11)	17.65 (12.86 to 23.32)	1.01 (0.60 to 1.76)	0.963
IV	143	115 (13)	28 (8)	19.58 (13.42 to 27.04)	1.13 (0.63 to 2.03)	0.674
V (least deprived/highest 20%)	138	114 (12)	24 (7)	17.39 (11.47 to 24.76)	1 (reference)	—
Missing	176	115	61	—	—	—
Ethnic group:†						
White	1090	729 (71)	361 (88)	33.11 (30.33 to 36.00)	1 (reference)	—
Asian, Asian British, or Asian Welsh	162	147 (14)	15 (4)	9.26 (5.28 to 14.81)	0.26 (0.14 to 0.44)	<0.001
Black, black British, black Welsh, or black Caribbean	130	106 (10)	24 (6)	18.46 (12.20 to 26.21)	0.56 (0.35 to 0.84)	0.003
Mixed or multiple ethnic groups/other‡	54	42 (4)	12 (3)	22.22 (12.04 to 35.60)	0.67 (0.34 to 1.19)	0.162
Missing	15	7	8	—	—	—

* Does not include women whose involvement with children's social care was not known (n=244).

† According to Office for National Statistics categories. Calculated rates do not include missing values.

‡ Office for National Statistics categories mixed or multiple ethnic groups were combined with other ethnic backgrounds because of small sample sizes.

CI, confidence interval; CSC, children's social care.

Women's medical, health, and pregnancy related characteristics were also compared (table 3). A higher proportion of women with CSC involvement had pre-existing medical problems (75% *v* 59%), mental health problems (75% *v* 27%), smoking during pregnancy (73% *v* 21%), and known substance misuse (55% *v* 5%) than women with no known CSC involvement. Of women with CSC involvement, 90% were multiparous, compared with 68% of those with no known CSC involvement. We found a small but significant difference in the proportion of women who received any antenatal care during pregnancy (89% *v* 94%, P=0.001), and among women who received antenatal care, a greater proportion of those with CSC involvement started antenatal care after 13 weeks' gestation (32% *v* 19%).

**Table 3:** Medical, health, and pregnancy related characteristics by children's social care involvement among women who died, 2014-22, UK*

Characteristics (n=1451 women who died)*	No known CSC involvement (n=1031)	Known CSC involvement (n=420)	P value between groups
**Medical and health characteristics**			
Pre-existing medical problems:			
Yes	600 (59)	304 (75)	<0.001
No	415 (41)	102 (25)	—
Missing	16	14	—
Mental health problems or psychiatric disorders:			
Yes	261 (27)	308 (75)	<0.001
No	718 (93)	100 (25)	—
Missing	52	12	—
Body mass index:			
Underweight <18.5	20 (2)	18 (5)	0.049
Normal 18.5-24	373 (38)	142 (37)	—
Overweight 25-29	247 (25)	102 (26)	—
Obese ≥30	347 (35)	125 (32)	
Missing	44	33	
Smoking during pregnancy:			
Yes	205 (21)	286 (73)	<0.001
No	769 (79)	108 (27)	—
Missing	57	26	—
Substance misuse:			
Yes	45 (5)	225 (55)	<0.001
No	966 (96)	181 (45)	—
Missing	20	14	—
**Pregnancy related characteristics**			
Parity:			
Nulliparous	292 (32)	37 (10)	<0.001
Multiparous	614 (68)	342 (90)	—
Missing	125	41	—
Multiple pregnancy:			
Yes	24 (2)	9 (2)	0.830
No	1007 (98)	411 (98)	—
Received any antenatal care:			
Yes	968 (94)	370 (89)	0.001
No	63 (6)	46 (11)	—
Missing	0	4	—
Antenatal care booked >13 weeks:†			
Yes	176 (19)	113 (32)	<0.001
No	770 (81)	238 (68)	—
Missing	22	19	—

Values are number (%). Calculated percentages do not include missing values.

P values are no known children's social care involvement group versus known children's social care involvement group.

* Does not include women whose involvement with children's social care was not known (n=244).

† Among women who received antenatal care.

CSC, children's social care.

Table 4 shows details of causes of death for the cohort. Women with CSC involvement had a higher proportion of deaths from suicide (20% *v* 10%), other mental health related causes, including substance overdose (30% *v* 3%), and homicide (5% *v* 2%) than women with no known CSC involvement. Women with CSC involvement had lower proportions of deaths for pre-existing conditions or conditions arising during pregnancy than women with no known CSC involvement, including cardiac causes of death (10% *v* 17%), cancer (5% *v* 20%), and neurological conditions (5% *v* 9%). Women with CSC involvement also had lower proportions of deaths from covid-19 infection than women with no known CSC involvement (1% *v* 4%). Although differences in the proportion of deaths in early pregnancy were not significant, CSC involvement is often triggered when women have their first antenatal contact in pregnancy. Therefore, CSC involvement might not have been started for many women who died in early pregnancy and were reported as unknown.

**Table 4:** Causes of death and children's social care involvement among women who died, 2014-22, UK*

Cause of death	No known CSC involvement (n=1031)	Known CSC involvement (n=420)	P value
Unintentional injury	29 (3)	14 (3)	0.596
Covid-19 disease†	46 (4)	5 (1)	0.002
Infection (excluding covid-19)	61 (6)	23 (5)	0.745
Cardiac	171 (17)	44 (10)	0.003
Deaths in early pregnancy	12 (1)	2 (<1)	0.224
Haemorrhage or amniotic fluid embolism	62 (6)	6 (1)	<0.001
Malignancy	201 (20)	20 (5)	<0.001
Neurology	91 (9)	23 (5)	0.031
Other indirect	87 (8)	22 (5)	0.036
Pre-eclampsia and eclampsia	17 (2)	3 (1)	0.166
Thrombosis and thromboembolism	89 (9)	34 (8)	0.739
Unascertained or other	15 (1)	2 (<1)	0.116
Suicide	97 (9)	82 (20)	<0.001
Other psychiatric causes	36 (3)	125 (30)	<0.001
Homicide	17 (2)	15 (5)	0.024

Values are number (%). Calculated percentages do not include missing values.

* Does not include women whose involvement with children's social care was not known (n=244).

† Only includes women who died in 2020-22.

CSC, children's social care.

Table 5 shows pregnancy and birth outcomes. Women with CSC involvement predominantly (75%) died in the late postnatal period (ie, six weeks to one year after the end of pregnancy). Compared with women with no known CSC involvement, women with CSC involvement were more likely to have a spontaneous vaginal birth (47% *v* 37%), with fewer maternal complications (9% *v* 14%) and critical care admissions (24% *v* 34%). We found no significant differences between the two groups for frequencies of stillbirths (9% *v* 9%) and neonatal deaths (4% *v* 5%).

**Table 5:** Pregnancy and birth outcomes by children's social care involvement among women who died, 2014-22, UK*

Characteristics (n=1451 women who died)*	No known CSC involvement (n=1031)	Known CSC involvement (n=420)	P value
**Pregnancy outcomes**			
Woman had not delivered a baby at death:			
Yes	137 (13)	58 (14)	0.792
No	894 (87)	362 (86)	
Timing of death:†			
Early (0-41 days after pregnancy)	401 (45)	91 (25)	<0.001
Late (42-365 days after pregnancy)	493 (55)	271 (75)	
Mode of birth (excluding early pregnancy losses):			
Spontaneous vaginal	306 (37)	149 (47)	0.007
Caesarean	458 (56)	154 (48)	
Other (including instrumental and vaginal breech)	60 (7)	15 (5)	
Missing	3	4	
Other maternal complication:			
Yes	146 (14)	35 (9)	0.003
No	872 (86)	376 (91)	
Missing	13	9	
Critical care admission:			
Yes	353 (34)	200 (24)	<0.001
No	678 (66)	330 (76)	
**Birth outcomes**			
Stillborn:‡§			
Yes	80 (9)	30 (9)	0.907
No	770 (91)	298 (91)	
Missing	1	2	
Baby died:¶‡§			
Yes	39 (5)	11 (4)	0.357
No	729 (95)	287 (96)	
Missing	2	0	

Values are number (%). Calculated percentages do not include missing values.

P values are no known children's social care involvement group versus known children's social care involvement group.

* Does not include women whose involvement with children's social care was not known (n=244).

† Among women who had given birth at the time of death. MBRRACE-UK (Mothers and Babies: Reducing Risk through Audits and Confidential Enquiries across the UK) distinguishes between early maternal deaths (ie, deaths occurring during or in the first six weeks after the end of pregnancy) and late maternal deaths (ie, deaths occurring between six weeks and up to one year after the end of pregnancy).

‡ Based on number of babies born >20 weeks’ gestation (n=851 in the no known children's social care involvement group and n=330 in the known children's social care involvement group).

§ Includes 30 multiple pregnancies (24 sets of twins and two sets of triplets).

¶ Among 770 live births in the no known children's social care involvement group and 298 in the known children's social care involvement group).

CSC, children's social care.

### Confidential enquiry into maternal deaths with children’s social services involvement

**Figure 3:**
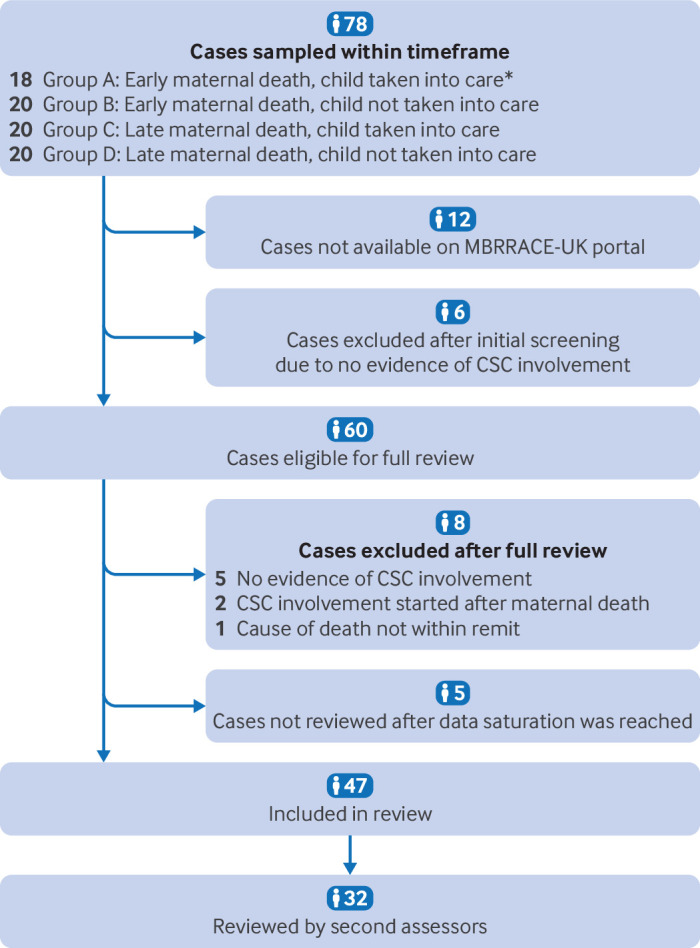
Flowchart of women who died in 2013-21 with children's social care involvement and whose medical notes were reviewed. *All cases available in this group. CSC=children's social care; MBRRACE-UK=Mothers and Babies: Reducing Risk through Audits and Confidential Enquiries across the UK

For our second objective, 47 sets of case notes were included in the confidential enquiry. Figure 3 shows the sampling, screening, and review process. Online supplemental table S5 gives the maternal and personal characteristics of the women whose case notes were included and frequencies of risk factors. The characteristics were largely comparable with the whole cohort.

Overall, women who were included in the case note review had a cumulative burden of complex social risk factors, with almost half having five or more complex social risk factors (21/47, 44.7%, online supplemental table S4). Two thirds of women reported domestic abuse from a partner or ex-partner (28/47, 59.6%), with similar numbers for substance misuse (27/47, 57.5%), homelessness and housing concerns (30/47, 63.8%), and childhood adversity (28/47, 59.6%). Referrals to CSC occurred mostly during the first trimester (33/47, 70.2%) and almost half of the women did not have their infant in their care (21/47, 44.7%). For multiparous women, almost two thirds of mothers did not have their older children in their care (24/36, 66.7%).

### Quality of maternity care

**Figure 4:**
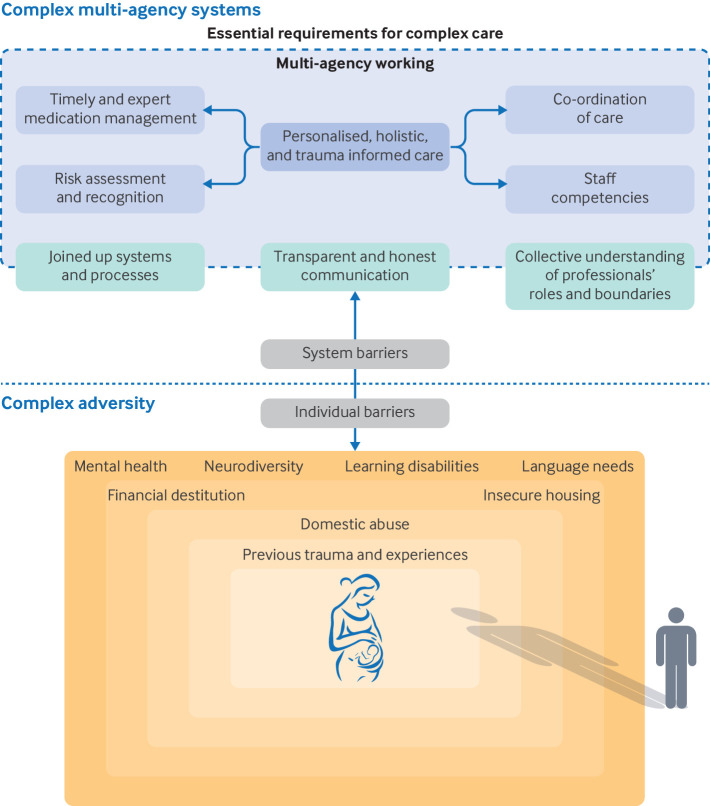
Visual display of confidential enquiry findings. Four essential requirements are required to provide personalised, holistic, and trauma informed care, in a complex multi-agency system. System complexity and complex adversity on an individual level create barriers to outreach, access, and engagement with care. The influence of perpetrators of domestic abuse (by a partner or ex-partner) extends across the individual barriers faced by women, even when the perpetrator is absent from clinical view

The confidential enquiry found some evidence indicating high quality, personalised care where professionals worked together to ensure the wellbeing and safety of mothers and babies. Four essential components to providing personalised, holistic, and trauma informed care for this cohort of women were identified. Figure 4 shows the principal findings of our case note review.

#### Risk assessment and recognition

Most women had some form of medical and obstetric complexity, along with their socioeconomic vulnerabilities. The interaction between medical and social complexity was often not explored by healthcare professionals, resulting in a single focused approach to risk assessment and recognition rather than a holistic approach. In some instances, risk management focused only on safeguarding the infant and did not always recognise the mother's own safeguarding needs. Alternatively, professionals focused entirely on medical risk factors, without exploring social circumstances. Conversely, social risk factors blinded professionals' abilities to recognise and manage underlying medical conditions, resulting in delayed treatment. The use of tick-box style medical risk assessment tools was not conducive for a biopsychosocial approach to risk and prioritised a medical notion of risk. Also, the wider social circumstances that affected engagement, treatment compliance, or self-monitoring of clinical warning signs were not consistently considered.

#### Timely and expert medication management

Access to appropriate and timely advice about drug treatments was challenging for many women, before conception and during the perinatal period. Women started their pregnancies with little information about the suitability of drug treatments during pregnancy or lactation, and with no guidance about ongoing titration or polypharmacology. Obtaining expert advice was often difficult, with drug treatment reviews sometimes carried out by clinicians with limited expertise in drug treatment management during the perinatal period. In some situations, women were advised against taking some psychotropic drug treatments that are considered safe during pregnancy or lactation, with detrimental consequences to their mental health. Also, sometimes women had substantial obstacles in securing repeat prescriptions, particularly in the postnatal period. For women receiving opiate substitution treatment, daily collection of their substitute drug treatment (most commonly methadone or buprenorphine) at their local pharmacist became challenging. In addition, limited professional understanding of harm reduction strategies and the importance of regular intake of substitute drug treatments caused delayed availability or administration during hospital admissions. The use of reductive labels such as "addict" or "drug user" were present in some of the women's records.

#### Coordination of care

For most women in the cohort, coexistence of complex social adversity and medical or obstetric comorbidity triggered a range of health and social care referrals and a sudden influx of professionals from various services and agencies during pregnancy. As a result, a high volume of antenatal appointments across different services were arranged. The care review showed that these appointments were often uncoordinated and with limited awareness by professionals of other services being involved in women's care. Sometimes this scheduling resulted in multiple appointments in different locations on the same day, or in close succession on subsequent days, adding to the burden of care for women. A so-called one-stop-shop approach was offered to only a few women, with input from different professionals at one specific time and location.

The care review found evidence indicating that most women were trying hard to attend this demanding schedule of antenatal appointments, which for several women was >30 different contacts during a pregnancy episode. For some women, however, uncoordinated appointment schedules became unattainable and resulted in non-attendance, or disengagement on occasion, that was often met with a punitive response through escalation to the social worker involved. This approach disregarded the overall levels of engagement that women had shown throughout their pregnancy care. Professionals were equally missing an overview of the services and professionals involved and at what time. This information would have provided coordination among the many professionals and unintentionally chaotic and burdensome appointment schedule.

#### Staff competencies

Healthcare professionals from various disciplines were involved in most women's care, but proactive safeguarding practice seemed to be the sole responsibility of (specialist) midwives. Obstetricians rarely reported relevant information about women's social circumstances during their clinical contact with women, and provided little to no input in the safeguarding process.

Some records had clear evidence of professional competency to explore sensitive issues, such as domestic abuse, substance misuse, and mental health difficulties, through judgment-free and transparent documentation of conversations, with referrals and signposting to relevant services. In most case notes, however, evidence of routine enquiry about sensitive issues was scant, and signposting to additional support was rarely seen. Our review found that even when maternity staff were aware of ongoing domestic abuse, most commonly through information sharing from other agencies, few women were signposted to, or supported by, relevant services.

In several instances, women presented with challenging or provocative behaviour, and clinical notes made in quick succession described an escalation of events. This reflected a heightened professional anxiety to manage and defuse such challenging situations. Documented evidence of professional recognition of the effect of trauma and its potential to be retriggered in maternity settings was rarely present in clinical notes, even though most women disclosed traumatic life events. Current guidelines to support trauma-informed care were generally not available or adopted during the timeframe of the study cohort (2014-22).^25^,^27^

### Multi-agency working and its challenges

One constant feature in the care of all the women included in the review was the wide range of services, agencies, and professionals involved. The complexity of a multi-agency system with various professionals was compounded by limited communication across and within services, uncertainty about professional roles, and disjointed processes, resulting in a rigid system unable to tailor care to the needs of women with complex social adversity. Different agencies and care providers often used different computer systems or health record systems, making it challenging to effectively and timely exchange important information. Processes for referrals and follow-up were not always clear, which affected communication between professionals. Also, professionals were working in high pressured environments and were sometimes expected to act outside the remit of their professional area of practice. These challenges often coincided with professionals being over reliant on others and not contributing equally to a collective approach in safeguarding women and their babies.

### Barriers to access and engagement

Mutual outreach, by women and professionals, was required for meaningful healthcare encounters and continuous engagement, but individual and systemic barriers were hindering this process. Almost all women had multiple social risk factors, and the cumulative burden of social adversity affected women's ability to access and engage with healthcare. The most common individual barriers to access and engagement were previous trauma and poor experiences of CSC involvement (including previous removal of older children), domestic abuse, financial hardship, insecure housing, and challenges related to mental health, neurodiversity, learning disabilities, and language needs (figure 4).

A common feature in all of the women's records was the absence of information about the identity of the unborn baby's biological father, partner, or ex-partner. This information is important in making maternity care more inclusive for fathers and partners, but is even more so in the context of domestic abuse when detailed information about the potential perpetrator is crucial for safety planning. Often, however, this information was entirely missing in women's clinical notes, causing confusion about the origin of the risk to women. Assessors felt that concerns about domestic abuse were often not dealt with by multiple agencies, resulting in missed opportunities for adequate signposting and support.

## Discussion

### Principal findings

In this study, among women who died during or in the year after the end of pregnancy, between 2014 and 2022, about one third had CSC involvement, and the proportion of women with CSC involvement has been steadily increasing in the past decade (figure 2). We found that CSC involvement was more likely among young, white, and unemployed women, and among those living in the most deprived areas. Medical and mental health comorbidity was more frequently seen in women with CSC involvement, and these women died more frequently from mental health related causes and homicide than women with no known CSC involvement. Most deaths in women with CSC involvement occurred during the late postnatal period (ie, six weeks to one year after the end of pregnancy).

The proportion of women with CSC involvement who died during the perinatal period was higher than previously reported because our study included surveillance data on late maternal deaths and coincidental deaths that were not included in standard maternal mortality analyses. The slight decrease in the last reporting period might be attributable to the lockdowns during the covid-19 pandemic when rates of safeguarding referrals to CSC were reduced by 10%.^28^

Inequalities in maternal and neonatal mortality and morbidity among black and Asian women have been widely reported in the UK and in other high income countries.^18 29^ These inequalities, however, were not found in our cohort. Findings from the surveillance data analysis showed that women with CSC involvement were predominately white, with women from black (rate ratio 0.56, 95% CI 0.35 to 0.84, P=0.003) and Asian (0.26, 0.14 to 0.44, P<0.001) backgrounds being significantly less likely to have CSC involvement than their white counterparts. This finding seemingly contradicts the existing evidence about racial inequalities in CSC involvement, which has previously described disproportionate representation of children from black and mixed ethnic groups within the UK's children's social services.^30 31^ More recent studies, however, have shown that although these inequalities exist, children from black and Asian families are referred at older ages than white children.^32^ These findings could explain why these ethnic and racial inequalities in CSC involvement had not yet manifested to the same extent during the period before birth or in the early postnatal stages. In addition, rates in our study were based on women who died and so might not reflect wider representation of those with CSC involvement. Also, ethnic inequalities in maternal mortality have been found for deaths during pregnancy and in the first six weeks after the end of pregnancy, with overall national maternity figures as the denominator. Our findings also showed that women with CSC involvement more frequently died during later stages of the postnatal period.

Our findings of socioeconomic inequalities in maternal mortality are similar to previous reports of increased mortality among women from the most deprived areas.^2 6^ Half of the women with CSC involvement were living in the most deprived areas in the UK, and many women whose records were reviewed had financial hardships and insecure housing. CSC involvement and, in particular, removal of infants into state care shortly after birth have been consistently linked with deprivation, with significant differences seen between the most deprived and affluent regions in the UK.^33 34^ Our confidential enquiry provided insight into the financial burden placed on women through accrued transportation costs because of the many community and hospital based appointments. The enduring effect of poverty on antenatal care was often not explored in clinical notes, and uncoordinated appointment schedules across a wide number of services became an additional challenge for women who already faced many disadvantages.

Domestic abuse was highly prevalent among women with CSC involvement (65%) compared with 3% in the group of women with no known CSC involvement. Unsurprisingly, twice the proportion of deaths from homicide was found in women with CSC involvement than in women with no known CSC involvement. Missing data on domestic abuse was a problem in both groups, and ample evidence exists indicating that domestic abuse is likely to be under-reported^35^,^37^ and not explored.^38^ Our confidential review benefitted from a retrospective viewpoint, meaning that at the time of the events, clinicians might not have had access to information or documents that were included in the MBRRACE-UK case notes and therefore might have been unaware of the full extent of the challenges for these women. Nevertheless, in notes from several women, we found evidence of clear indications of abusive or controlling behaviour or that such information had been shared with maternity staff (eg, through the sharing of police reports or admissions to emergency services with assault injuries). The risk of domestic abuse was not recognised by professionals in either of these situations, and there was no documentation in clinical notes that indicated this risk had been fully explored with women. The absence of such documentation can be attributed to secret recording systems in handheld notes, to ensure women's safety, but even with the varied clinical expertise among assessors, no evidence of explorative conversations of domestic abuse could be detected. This finding reflects a more general limited professional awareness of the cumulative burden of social adversity on maternal outcomes,^39^,^41^ women's mental health,^42^ maladaptive coping strategies,^43^ and overall availability to engage with perinatal healthcare.^44^ A consistent holistic approach to women's biopsychosocial presentations, adopted by all professionals, is required to acknowledge and recognise the effect of complex social adversity on women's mental and physical wellbeing.

### Strengths and limitations of this study

To our knowledge, our study is the first UK-based study using both national surveillance data and confidential enquiry methods to investigate the care of women with CSC involvement who subsequently died. A major strength of our study was the use of national surveillance data, allowing our analysis to include all women who died in the UK over an eight year period. Another strength was the use of confidential enquiry methodology; data on an individual are aggregated to show common factors to identify areas where remedial action might be possible to save lives.^45^ Using data from healthcare records also minimises the risk of recall bias, and our approach in forming the cohort reduced any potential selection bias.

Our study had some limitations. Our national maternal mortality surveillance data analysis was limited by missing data for some demographic variables. Information about specific sociodemographic items might not be recorded in the clinical record, which limits the ability of the professional in the maternity services tasked to complete the MBRRACE-UK surveillance form. This challenge is particularly relevant for those women who died before becoming known to maternity services because routine booking questions were never asked. Current guidelines are inconclusive about the range and frequency of inquiry about specific social risk factors, such as housing or history of child abuse, or are known to be poorly adhered to, such as routine inquiries about domestic abuse. These shortcomings can further compound missingness for these sociodemographic covariates within our study.

A further challenge was the absence of comparable UK data for CSC involvement in the wider pregnant and postnatal population. At present, the Office for National Statistics' Child in Need census only captures data on CSC involvement from local authorities in England and Wales, although in separate datasets. Data from the Office for National Statistics on live births include pregnancies and births in England and Wales, with births in Scotland registered by Public Health Scotland. This fragmentation of overall UK data across the four UK nations, whether for pregnancies, live births, or CSC involvement, limited the comparisons we could draw between our MBRRACE-UK cohort of women who died during or after pregnancy, including those with CSC involvement, and the wider UK cohort of women with a pregnancy or birth in this context. Therefore, we cannot state how the cohort of women with CSC involvement who subsequently died is representative of the wider cohort of women with CSC involvement during pregnancy or the year after birth. Hence our findings might not be generalisable to the wider population of women and children with CSC involvement during pregnancy or before their child's first birthday.

The confidential enquiry sample was 11% of the entire cohort of women who died during 2014-22 with CSC involvement. Similar concerns about missing data, as described above, were a result of inconsistent documentation on social risk factors in maternity notes. Because minimal discrepancies were identified in <5% of data extraction fields, and to reduce the potential trauma of cumulative exposure to case notes,^46^ only two thirds of the sample were reviewed twice. It is important to note that causality cannot be inferred from this observational analysis.

Confidential enquiry methods are limited by what is documented in clinical records, by healthcare professionals working in a clinical system that is under strain. Illegible handwriting, missing pages, or redacting of identifiable information sometimes complicated interpretation and data extraction. There may be important additional nuances of care such as compassion, non-verbal communication and biased attitudes that were undocumented. WHO MDSR methodology recommends the use of reviewers with expertise to identify both non-medical and medical factors in care.^17^ Our methods for this review were robust, and members with lived experience and the wellbeing of assessors were carefully considered, with access to regular supervision from experienced assessors and peer support. It should be recognised that the findings will be grounded in the multiple domains of expertise of the assessors who contributed.

### Conclusions

To our knowledge, this study is the first of its kind in the UK to analyse maternal death surveillance data with a focus on CSC involvement, and retrospectively review the care records of a sample of women in this cohort. We believe our findings substantially enhance our understanding of the complex care journeys that women navigate during pregnancy and the postnatal period. We identified four essential components to providing holistic and personalised care when dealing with social and medical complexity and evidenced how access and engagement is hindered by the cumulative burden of social risk factors as well as by the complexities of a multi-agency system. While some women received excellent, coordinated care, we identified that urgent changes to practice, clinical guidance, and policy are required to prioritise this group of marginalised women. Essential to this process is an integrated and coordinated multidisciplinary approach by health, social care, and the criminal justice system, to adapt maternity care, and by extension perinatal healthcare, for the complex physical, mental, and social needs of this under-served group of women. These changes also need to be implemented within the current resource and service constraints.

## Supplementary material

10.1136/bmjmed-2025-001464 online supplemental file 1

## Data Availability

Data are available upon reasonable request.
