# Supplementary material for: Characteristics, outcomes, and maternity care experiences of women with children’s social care involvement who subsequently died: national cohort study and confidential enquiry
Source: BMJ Med. 2025 Jul 10;4(1):e001464. doi: 10.1136/bmjmed-2025-001464 (PMC12258268; doi:10.1136/bmjmed-2025-001464)
Supplement: online supplemental file 1 [file bmjmed-4-1-s001.pdf]

Supplementary materials

Supplementary Table S1 Complex social risk factors and key areas of good care to guide data extraction

Complex social risk factors	Key areas of good care
Domestic abuse<br>Substance misuse<br>Mental Health issues<br>Criminal justice involvement<br>Homelessness/Insecure housing<br>Young (<20yrs)<br>Learning difficulties or disability<br>Physical disabilities<br>Absence of social support (partner/family)<br>Significant financial need<br>Recent migrant (<1yr)<br>Unable to speak or understand English<br>Care experienced (as child/adolescent)<br>Concerns regarding partner or father of infant	1. Women should have access to all relevant services as early as possible, with clear communication between agencies<br>2. Women should receive support that is specialist and continuous during pregnancy, birth and early motherhood<br>3. Women should receive support that is women-centred, holistic and culturally appropriate<br>4. Women should receive support that is trauma-informed and trauma-responsive<br>5. Women should receive support that is responsive to their specific needs, including mental health support?<br>6. Women should be having their birth preferences and parenting choices respected<br>7. Women should have their rights upheld through clear ways to express concerns, challenge inaccuracies and make complaints about unfair or poor practice<br>8. Was there any evidence of judgement or bias in care or support received

Supplementary Table S2 Maternal Death Case Note Review when Social Services are involved

**Case ID**				
FROM DATA SURVEILLACE FORM	**Cause and place of death**			
	**Early or late maternal death**	Pregnancy (gestation):		
		Postnatal (w/m PN):		
	**Summary of case and care** (only to be used as prompts to identify possible vignettes and demonstrate key findings)			
	Demographics	Age at death		
		Ethnicity		
		Country of birth		
		Socio-economic status (IMD quintile)		
		Employment status		
		Partner status e.g. single or has a partner/spouse		
		BMI at booking		
		Gestational age at booking		
	**Medical/obstetric risk factors**		**Yes/No**	**Detail**
	Medical history	Gravidity & Parity		
		History of stillbirth/Neonatal death/SIDS		
		Known medical conditions		
		Known obstetric risk factors		
		Known mental illness		
**Complex social factors of multiple disadvantage***			**Present (Y/N/NA)****	**Actions taken/support offered/support in place**
Complex social risk factors	Domestic abuse (If possible provide information about perpetrator and type of abuse)			
	Substance misuse (please specify: alcohol/illicit drugs/over the counter or prescription drugs used in harmful way)			
	Mental Health issues			
	Criminal justice involvement			
	Homelessness/Insecure housing			
	Young (<20yrs)			
	Learning difficulties or disability			
	Physical disabilities			
	Absence of social support (partner/family)			
	Significant financial need			
	Recent migrant (<1yr)			
	Unable to speak or understand English			
	Care experienced (as child/adolescent)			
	Concerns re partner (such as paternal substance misuse/MH/CJS)			
	Other			
**Summary of social services involvement** (current pregnancy and any previous contact)				
Social Services involvement	Circumstances of referral (if known)<br>- Referrer<br>- Time of referral<br>- Main reason for referral<br>- Length of time between referral and actual involvement from Social Services<br>- Any referrals rejected			
	Involvement for: 1) Mother; 2) Baby; 3) Both			
	Involvement prior to death (Yes/No)			
	Highest level of involvement: (CIN/CP/PLO/ICO/PPO/s20/other)			
	Child taken into care (Yes/No) – if yes, please give details if this was prior or after maternal death			
	Previous involvement, incl. removals (Yes/No) – if yes, please give details			

Thematic Guidance for Case Note Review with prompts***

1. Women should have access to all relevant services as early as possible, with clear communication between agencies
(BC Charter Principle 1, 4 & 6)

- Was there a confident and skilled conversation about potential safeguarding concerns at booking?
- Was there a prompt referral to LA as soon as safeguarding concerns were identified?
- Was there evidence or clear documentation of thresholds when referrals were sent?
- Was there a timely offer of specialist support (1st trim)?
- Were professionals' concerns and plans shared with parents, while continually checking parents' understanding?
- Was there consistent and frequent multi-agency communication?
- Were services joined-up, co-located where possible?
- Did the woman have access to all relevant universal and specialist services required?
- Was the woman offered or signposted to antenatal education classes?
- Was the woman excluded from any services/care on the grounds of social services involvement? If so, which services?
- Was there any evidence of MDT safety planning prior to discharge for women subject to Domestic Abuse?

2. Women should receive support that is specialist and continuous during pregnancy, birth and early motherhood (BC Charter principles 1)

- Was there involvement of staff with expertise in safeguarding?
- Were there any perinatal mental health practitioners involved?
- Was the woman seen in a specialist pathway for women with social services involvement?
- Was there evidence of flexibility in appointments?
- Was there evidence of professional understanding of risk, for instance re coercive control.
- Was the woman prioritised for Continuity of Carer?
- If fragmented care, give summary or examples
- In case of domestic abuse, did she have access to appropriate support, such as an IDVA?
- In case of substance misuse, did she have access to specialist addiction services?
- Was there an assertive outreach approach taken to the woman's postnatal care?
- Was the postnatal offer extended beyond 10days PN?
- Was information shared clearly with GP, health visitors and other services involved?

3. Women should receive support that is women-centred, holistic and culturally appropriate (BC Charter principle 2 & 9)

- Was there any consideration given to the woman's specific learning and communication needs or preferences (incl. interpreter requirements)?
- Was there evidence of parental distrust of services? If so, what actions did maternity staff undertake to mitigate this?
- Was care oriented towards the baby and not towards the mother?
- Did the woman have opportunities to discuss issues without partner present?
- Was the mother's privacy, dignity and confidentiality respected?
- Was supervision proportionate to the level of risk and concern?
- Was the mother supported to attend the court hearing, especially if still recovering in hospital?

4. Women should receive support that is trauma-informed and trauma-responsive (BC Charter principles 3)

- Is there evidence that the impact of previous social services involvement or previous removals was considered?
- Was the mother's history of trauma taken into account?
- Was challenging or avoidant behaviour viewed through a lens of possible past trauma?
- Did the woman experience any form of racism?
- Did the woman experience any discrimination related to protected characteristics?
- Were efforts made to minimise or mitigate the risk of re-triggering trauma?
- In case of domestic abuse, was there evidence of victim-blaming (i.e. women being held accountable for partner's behaviour?)
- Was sensitivity shown regarding the information shared with other professionals/in records and its potential impact on parents?

5. Women should receive support that is responsive to their specific needs, including mental health support? (BC Charter principles 5, 7, 9 and 10)

- Were referrals due to MH concerns made at the earliest opportunity?
- Was the woman supported by MH professionals to understand any MH diagnoses?
- Did she have access to appropriate MH provisions?
- Were transitions between MH services based on need?
- Was there a protected postpartum period immediately after birth to allow for recovery and bonding?
- If baby was on NICU, was access to baby equivalent to other mothers without SSI?
- Were the immediate basic and emotional needs of the mother prior to hospital discharge checked?
- Was community support put in place prior to hospital discharge?
- Was there access to services and professional support post-removal?
- If no longer eligible post-removal, was there timely alternative support made available?
- Was the woman prioritised to ensure timely offer of MH support post separation?

6. Women should be having their birth preferences and parenting choices respected (BC Charter Principles 8 and 10)

- Were LA plans re birth arrangements and plan for baby shared by 30w gestation?<br>- Were parents involved in producing the birth arrangements, incl. choice of birthing partner?<br>- Were women's preferences around birth and early PN period discussed and documented?<br>- Were women's preferences around birth and early PN period followed?<br>- Was she supported by a birth partner?<br>- Was the mother given maximum opportunities to parent her baby, in the way she preferred (e.g. feeding choices, skin-to-skin)?<br>- Was there any consideration given that the woman needed space and time to prepare emotionally and practically for a possible separation?<br>- Was there any consideration for continuing post-separation bonding (eg. creating mementos etc)?<br>Was mother given clear information about future contact arrangements prior to separation?
**7. Women should have their rights upheld through clear ways to express concerns, challenge inaccuracies and make complaints about unfair or poor practice (BC Charter Principle 14)**
- Did the woman challenge any information collected or presented due to inaccuracy?<br>- Was there any information shared re complaints procedures?<br>- Was she supported by maternity staff to raise concerns with other members of the multi-professional team around her?<br>Did the woman have access to an advocate or some independent support
**8. Additional: Was there any evidence of judgement or bias in care or support received?**
Please provide any examples of language indicative of judgement or bias related to social services involvement or complex social factors

* Cosstick et al., 2022: doi: 10.1016/j.eclinm.2022.101587; Birth Companions 2019 report

** Yes: asked and present / No: asked but not present / NA: not asked or no information available

*** Guidance taken from Birth Companions Charter for women with involvement from children's social care (2023) and Born Into Care best practice guidance for when the state intervenes at birth (2023)

Supplementary Table S3 Prevalence of missingness on Children Social Care involvement per triennium reporting period

Triennia	Missing data All (%)	Missing Early maternal death (%)	Missing Late maternal death (%)
2014-16	17.39%	3.8%	28.85%
2015-17	17.12%	8.05%	23.96%
2016-18	13.71%	8.26%	18.03%
2017-19	13.13%	12.8%	13.38%
2018-20	8.21%	9.76%	6.9%
2019-21	9.25%	14.12%	5.14%
2020-22	12.64%	15.54%	10.03%

Supplementary Table S4 Prevalence of missing data on variables

**Total N=1,451⁺**	**Missing data Total (%)**	**Missing* No known CSC involvement N= 1,031 Frequency (%)**	**Missing* Known CSC involvement N=420 Frequency (%)**
**Socio-demographic characteristics**			
Partner	17 (1.2%)	7 (0.7%)	10 (2.4%)
Socioeconomic status	215 (14.8%)	118 (11.4%)	97 (23.1%)
Living arrangements	114 (7.9%)	38 (3.7%)	76 (18.1%)
Domestic abuse	378 (26.1%)	272 (26.4%)	106 (25.2%)
History of abuse as a child	841 (58.0%)	543 (52.7%)	271 (64.5%)
Social deprivation (IMD)	176 (12.1%)	115 (11.2%)	61 (14.5%)
Ethnic group	15 (1.0%)	7 (0.7%)	8 (1.9%)
**Medical, health and pregnancy characteristics**			
Pre-existing medical problems	30 (2.1%)	16 (1.6%)	14 (3.3%)
Mental health problems	64 (4.4%)	52 (5.0%)	12 (2.9%)
BMI	77 (5.3%)	44 (4.3%)	33 (7.9%)
Smoking during pregnancy	83 (5.7%)	57 (5.5%)	26 (6.2%)
Substance use	34 (2.3%)	20 (1.9%)	14 (3.3%)
Parity	166 (11.4%)	125 (12.1%)	41 (9.8%)
Received antenatal care	4 (0.3%)	0	4 (1.0%)
Antenatal care booked >13wks	41 (2.8%)	22 (2.1%)	19 (4.5%)
**Pregnancy and baby outcomes**			
Mode of birth	7 (0.5%)	3 (0.3%)	4 (1.0%)
Other maternal complication	22 (1.5%)	13 (1.3%)	9 (2.1%)
Stillborn	3 (0.2%)	1 (0.1%)	2 (0.5%)
Baby died	2 (0.1%)	2 (0.2%)	0

*does not include women whose CSC involvement status was unknown (N=244)

*Missingness within each group (No known CSC involvement and Known CSC involvement) is given as frequency and proportion within each group. This allows for comparison of missingness between both groups.

Supplementary Table S5 Maternal characteristics of the women included in the confidential enquiry

**Demographic characteristics**	**Sampled cohort n (%)**
**Ethnicity**	
White	39 (83.0%)
Asian/Asian British/Asian Welsh	3 (6.4%)
Black/Black British/Black Welsh/African	1 (2.1%)
Mixed or multiple ethnic groups	1 (2.1%)
Other Ethnic group	1 (2.1%)
Missing	2 (4.3%)
**Age at time of death M_{Age} 30.4, Range 16-41**	
Under 20y	3 (6.4%)
20-24y	8 (17.0%)
25-39y	34 (72.3%)
40y or older	2 (4.3%)
**Parity at time of death**	
P0	3 (6.4%)
P1	11 (23.4%)
P2	14 (29.8%)
P3 or more	19 (40.5%)
**Time of death**	
Pregnancy	10 (21.3%)
Early maternal death <6wks	13 (27.7%)
Late maternal death >6wks	24 (51.1%)
**Relationship status**	
Single	7 (14.9%)
In relationship	32 (68.1%)
Married	4 (8.5%)
Separating during the perinatal period	4 (8.5%)
**Living arrangements**	
Living alone	13 (27.7%)
Living with partner, parents or extended family	22 (46.8%))
Unstable housing situation (incl. in custody & supported living)	7 14.9%)
Missing	5 (10.6%)
**Employment status**	
Unemployed[+]	39 (83.0%)
Housewife/looking after family	4 (8.5%)
Missing	4 (8.5%)
**Clinical characteristics**	
Late booking (>13weeks gestational age)	16 (34.0%)
Known medical risk factors	35 (74.5%)
Known obstetric risk factors	39 (83.0%)
Known mental illness	39 (83.0%)
**Social complexity and social care involvement**	
Five or more complex social risk factors*	21 (44.7%)
Domestic abuse during pregnancy or the postnatal period	28 (59.6%)
Substance misuse during pregnancy or the postnatal period	27 (57.5%)
Homelessness or insecure housing	30 (63.8%)
**Referred to social services for**	
Mother	1 (2.1%)
Unborn or infant	39 (82.9%)
Both	6 (12.8%)
Older children	1 (2.1%)
Referred in first trimester	33 (70.2%)
Infant taken into care	21 (44.7%)
Previous child(ren) not in parental care (if multiparous, n=36)	24 (66.7%)

[+] Includes being in custody, permanently sick or disabled, in education, no rights to work

* Risk factors included: Perinatal domestic abuse, perinatal mental health issues, perinatal substance misuse, CJS involvement during the perinatal period, homeless or insecure housing, young age (20y or younger at time of death), learning difficulties, physical disabilities, absence of social support, significant financial need, recent migrant (less than 1yr), unable to speak or understand English, care-leaver, significant Adverse Childhood Experiences)
