# Supplementary material for: Characteristics, outcomes, and maternity care experiences of women with children’s social care involvement who subsequently died: national cohort study and confidential enquiry
Source: BMJ Med. 2025 Jul 10;4(1):e001464. doi: 10.1136/bmjmed-2025-001464 (PMC12258268; doi:10.1136/bmjmed-2025-001464)
Supplement: Supplementary file 2 [file bmjmed-2025-001464.reviewer_comments.pdf]

PEER REVIEW HISTORY

BMJ Medicine publishes all reviews undertaken for accepted manuscripts. Reviewers are asked to complete a checklist review form and are provided with free text boxes to elaborate on their assessment. These free text comments are reproduced below.

ARTICLE DETAILS

**TITLE (PROVISIONAL)**	Characteristics, outcomes and maternity care experiences of women with Children's Social Care involvement who subsequently died: A national cohort study and confidential enquiry
**AUTHORS**	De Backer, Kaat; Felker, Allison; Rose, Emma; Bull, Caroline; Labisi, Oluwaseun; Kitchen, Kirsty; Mason, Claire; Montgomery, Elsa; Sandall, Jane; Easter, Abigail; Knight, Marian; Vousden, Nicola

VERSION 1 - REVIEW

**REVIEWER NAME**	Goodfellow, Laura
**REVIEWER AFFILIATION**	University of Liverpool, Women's and Children's Health
**REVIEWER CONFLICT OF INTEREST**	None
**AI DISCLOSURE**	No
**DATE REVIEW RETURNED**	14-Mar-2025

**GENERAL COMMENTS**	Thank you for the opportunity to review this manuscript. This is an important paper focusing on maternal mortality with/without social care involvement, and exploring the quality of care of women who died who had social care involvement.<br><br>I think this is a well conducted and thorough study that would benefit from publication in BMJ Medicine. I especially like figure 4 and I think this will be helpful for practitioners. I only have minor comments.<br><br>Minor points<br>• I'm struggling to follow the language on p7 lines 18-23- this states "This is nearly a two-fold increase from the 12% in the 2012-2014 triennium (3), and comparable with figures from the Office of National Statistics of infants known to CSC.(4)". What is comparable with the ONS figures- have the overall numbers of pregnancies with CSC involvement increased so dramatically (I don't think so) or do the ONS statistics recognise an increase in deaths of mothers with CSC involvement- this also seems odd? I suspect this is my misunderstanding. It would be helpful to know the proportion of CSC involvement in pregnancies in the same period in either the background population, or in women who did not die during the same period to contextualise the level of involvement with women that died.<br>• I'm struggling to understand the right column of table S1. It states women 'should' receive certain elements of care. Whilst these all seem appropriate elements of care that should be received I do not understand where these themes arose from- were they the standards that you were judging care against- and if so can you reference those? I think they were developed from a multi-

	disciplinary steering group and lived experience panel- but it would be good to understand more about that process? The labelling of the right side of table S1 as ‘themes’ initially made me think that this related to information developed from the data, but I think I mis-understood this.<br>• P 4 line 43- should this ‘was’ be ‘were’? “subset of 47 women’s healthcare records was included in the confidential enquiry.”<br>• The base of table 3 seems to be missing some yes/no responses

**REVIEWER NAME**	Cowley, Laura
**REVIEWER AFFILIATION**	Swansea University Medical School, Data Science
**REVIEWER CONFLICT OF INTEREST**	None
**AI DISCLOSURE**	No
**DATE REVIEW RETURNED**	26-Mar-2025

**GENERAL COMMENTS**	This is a brilliant paper, and was a pleasure to read, despite the sombre topic area.<br>I just have a couple of minor comments.<br>Abstract: I would say "proportion of women with Children's Social Care (CSC) involvement who died...."<br>The section "What is already known on this topic" is too similar to the background section of the Abstract, consider rewording one of the sections to avoid repetition. Introduction - spell out/define what MBBRACE is at first use. Last line of page 6 going into first line of page 7 is not quite grammatically correct. Typo on page 8 line 43.<br>Stat methods: it would be useful to include a few lines about why the chi square test was the most appropriate test to use for your research questions. For example, if you had performed logistic regression analyses, you could have demonstrated the most important variables that predicted maternal death in CSC-involved women after controlling for everything else. I am curious as to why this wasn't done.

**REVIEWER NAME**	Harron, Katie
**REVIEWER AFFILIATION**	
**REVIEWER CONFLICT OF INTEREST**	None
**AI DISCLOSURE**	No
**DATE REVIEW RETURNED**	01-Apr-2025

**GENERAL COMMENTS**	This is a really interesting and important paper, on a group of mothers who are not typically included in research. Overall it is written very clearly, but I have a couple of queries.<br><br>My main concern is the level of missing data. I realise that not much can be done about this, and it doesn’t necessarily invalidate the findings, but it would be helpful to explore the mechanisms for this. For example in Figure 2, you can see that the increase in the percentage of mothers with CSC involvement recorded coincides with a decrease in missing data. So – we cannot say whether this apparent increase is an actual increase, or just improved recording over time. The authors do state that there has been a steady increase since 2014, but this was not tested statistically. Can the authors comments on this?

	Secondly, there is a lot of missing data on the socio-demographic and clinical characteristics of women who died. This varies according to the variable – was it the case that a specific group of women had very little information recorded across all variables, or was the missingness more random? Can you say anything about the characteristics of mothers who were most likely to have missing data and whether these were more likely those with CSC involvement or not?<br><br>Although some limitations of the case note review and given, there needs to be a more thorough exploration of the limitations of the study within the discussion (including the surveillance data element).<br><br>Abstract results – please define what age range is meant by "younger", and what the comparator is. The methods of statistical analysis used to derive the relative risks should be stated in the design section, and RR should be given in full the first time it is used. Similarly, the comparator group for ethnicity (white?) and deprivation (is this all other groups, or least deprived?) needs to be stated.<br><br>Please give the percentage of women who were excluded because of a lack of information on the surveillance form relating to CSC involvement. How representative were those who were excluded for this reason?<br><br>Is there a justification for grouping ethnicity into the 5 broad categories? If so, this should be given.<br><br>Rather than stating that you used the 'iri' command, please describe how this command calculates the mortality rate ratio.<br><br>Table 2 – how the "rate" column has been labelled is a little confusing. Rather than a rate, I think this is just the percentage of women who died, who had CSC involvement. Suggest this is relabelled.<br><br>Table 3 – above substance use and below BMI, there is a row title missing. Received any antenatal care – row titles missing.<br><br>Figure 2 – typo "naternal"<br><br>Supplementary table S2 is interesting. Do you have data on the non-sampled cases that can readily be extracted, so that we can see how representative the sample is?

VERSION 1 – AUTHOR RESPONSE

Reviewer 1	
Thank you for the opportunity to review this manuscript. This is an important paper focusing on maternal mortality with/without social care involvement, and exploring the quality of care of women who died who had social care	Thank-you for your positive feedback to our manuscript. We are grateful for your suggested revisions and hope our changes have further strengthened our manuscript.

involvement. I think this is a well conducted and thorough study that would benefit from publication in BMJ Medicine. I especially like figure 4 and I think this will be helpful for practitioners.	
I'm struggling to follow the language on p7 lines 18-23- this states "This is nearly a two-fold increase from the 12% in the 2012-2014 triennium (3), and comparable with figures from the Office of National Statistics of infants known to CSC.(4)". What is comparable with the ONS figures- have the overall numbers of pregnancies with CSC involvement increased so dramatically (I don't think so) or do the ONS statistics recognise an increase in deaths of mothers with CSC involvement- this also seems odd? I suspect this is my misunderstanding. It would be helpful to know the proportion of CSC involvement in pregnancies in the same period in either the background population, or in women who did not die during the same period to contextualise the level of involvement with women that died.	Thank-you for this comment. We made the following change.<br><br>*This is nearly a two-fold increase from the 12% in the 2012-2014 triennium (3). A similar trend of rising rates of CSC involvement during pregnancy and among infants under the age of one can be found in England through the Child In Need Census data from the Office of National Statistics. (4)*<br><br>To highlight the extent of the issue further, we have also included the following sentence:<br><br>*Recent work using the CIN Census data estimated that by age 18 one in four children in England are identified by CSC as needing support at some point. (5)*<br><br>Unfortunately, there is no national data to compare the MBRRACE-cohort with the wider UK cohort of pregnant women and women who gave birth in the same four nations and who have CSC involvement during their pregnancy or the year after birth. ONS Child in Need Census data is limited to England and Wales, albeit separately. So are the ONS data on live births. This makes it difficult to create a denominator to investigate the actual proportion of women in the four nations who were pregnant or gave birth for the same time period, and how many of those were involved with CSC.
I'm struggling to understand the right column of table S1. It states women 'should' receive certain elements of care. Whilst these all seem appropriate elements of care that should be received I do not understand where these themes arose from- were they the standards that you were judging care against- and if so can you reference those? I think they were developed from a multi-disciplinary steering group and lived experience panel- but it would be good to understand more about that process? The labelling of the right side of table S1 as 'themes' initially made me think that this	Thank-you for your comment. The 'themes' in S1 were extracted from two reports that have been recently published: The 'Born Into Care Best Guidance for when the State intervenes at birth' and the 'Birth Charter for women with involvement from Children's Social Care'. Both have been developed through extensive lived experience involvement. We distilled the themes from both documents and identified seven common or overlapping themes. These were used as a template to guide the thematic

related to information developed from the data, but I think I mis-understood this.	analysis of quality of maternity care when reviewing the case notes.<br><br>We understand the use of 'themes' is confusing in this context, and could imply results, rather than the 'thematic framework' we designed to guide the case note review. We have therefore replaced this with the following in the manuscript:<br><br>*The template also contained key areas of good care for women with CSC involvement*.<br><br>The rest of that section already contains detailed information about the two documents, how they were developed and what include.<br><br>In the table, we have changed the caption and the heading to:<br><br>*Key areas of good care to guide data extraction*
P 4 line 43- should this 'was' be 'were'? "subset of 47 women's healthcare records was included in the confidential enquiry."	Thank-you. We have changed this in the revised manuscript
The base of table 3 seems to be missing some yes/no responses	It seems some issues regarding table formatting occurred when uploading them on the portal. The tables have now been embedded in the main manuscript and this should have resolved the issue.

**Reviewer 2**	
This is a brilliant paper, and was a pleasure to read, despite the sombre topic area.	Thank-you for such positive feedback on our manuscript. We are grateful for your suggested revisions and hope our changes have have further strengthened our manuscript.
I would say "proportion of women with Children's Social Care (CSC) involvement who died...."	We have made this change in the revised manuscript.
The section "What is already known on this topic" is too similar to the background section of the Abstract, consider rewording one of the sections to avoid repetition.	Thank-you for this suggestion. We have now reworded this in the section "What is already known on this subject?" as follows:<br><br>*Children's Social Care (CSC) involvement during pregnancy and among infants under the age of one has increased in the last decade. Previous confidential enquiries into maternal deaths in the UK have described how women with multiple adversity face many biases,*

	impacting the quality of maternity care they receive. A proportion of these women will also have CSC involvement, and for some this may result in removal of their infant from their care. CSC involvement during pregnancy and the postnatal period has been referred to as one of the most challenging aspects of contemporary clinical maternity practice and is known to be associated with increased maternal mortality and morbidity.
Introduction - spell out/define what MBBRACE is at first use.	We have added this in the revised manuscript.
Last line of page 6 going into first line of page 7 is not quite grammatically correct.	We have corrected this now.
Typo on page 8 line 43.	Apologies, in our copy there is no line number 43 on page 8. I checked if this might have been line 143 but there do not seem to be any typos in this line.
Stat methods: it would be useful to include a few lines about why the chi square test was the most appropriate test to use for your research questions. For example, if you had performed logistic regression analyses, you could have demonstrated the most important variables that predicted maternal death in CSC-involved women after controlling for everything else. I am curious as to why this wasn't done.	Since this is a rarely studied cohort, the aim of this study was to explore and describe the characteristics in a cohort of women. We therefore focused on descriptive statistical analyses, where the most appropriate test was the chi square test. Our focus was not to predict maternal death in CSC-involved women given the presence of any particular variable. Also, we do not have comparable data for women who did not die, therefore it was not possible to investigate maternal death as an outcome given CSC involvement as an exposure.<br><br>We have included an additional section on our decision-making in the main manuscript too:<br><br>*For the first objective, descriptive statistical methods were used to investigate socio-demographic, clinical, social and pregnancy-related characteristics and causes of death.*

**Reviewer 3**	
This is a really interesting and important paper, on a group of mothers who are not typically included in research. Overall it is written very clearly, but I have a couple of queries.	Thank-you for your positive feedback to our manuscript. We are grateful for your queries and suggested revisions and hope our changes have further strengthened our manuscript.
My main concern is the level of missing data. I realise that not much can be done about this,	We want to make it clear that, similar to the data in Table 1 to 5, the proportions of women who

and it doesn't necessarily invalidate the findings, but it would be helpful to explore the mechanisms for this. For example in Figure 2, you can see that the increase in the percentage of mothers with CSC involvement recorded coincides with a decrease in missing data. So – we cannot say whether this apparent increase is an actual increase, or just improved recording over time.	died with CSC involvement in each overlapping triennia are women known to have CSC involvement and women who were known to not have CSC involvement. For some women, neither box on the surveillance form was ticked, for instance because they died in early pregnancy before Children's Social Care might have become involved.<br><br>We agree the title and key on the graph was misleading in this respect as it does not represent missing data as such, but the proportion of people who were not known to have CSC involvement. We have updated the figure to make this clearer. This also means the observed increase in the proportion is independent of the missing values.
The authors do state that there has been a steady increase since 2014, but this was not tested statistically. Can the authors comments on this?	Thank you for this comment. The steady increase was based on observed proportions without statistical analyses but we have since applied Stata 17 command 'nptrend', to the data, which performs four different non-parametric tests for trend. Based on this analysis there is a statically non-significant increase in the proportion of women who died during pregnancy or up to 6 weeks after (early deaths) with CSC involvement (p=0.060) and a statistically significant increase in the proportion of late deaths (between 6 weeks and 1 year after the end of pregnancy) with CSC involvement (p=0.019). The overall proportion (early and late) was also statistically significantly increased (p=0.019).
Secondly, there is a lot of missing data on the socio-demographic and clinical characteristics of women who died. This varies according to the variable – was it the case that a specific group of women had very little information recorded across all variables, or was the missingness more random? Can you say anything about the characteristics of mothers who were most likely to have missing data and whether these were more likely those with CSC involvement or not?	We recognise that missing data is a limitation of the dataset and have added a comment in the discussion to this extent. The high proportion of missing information regarding social risk factors is consistently highlighted in MBRRACE reports and the potential underestimation of the proportion of women who died with social service involvement is also noted. Due to efforts by the MBRRACE team to find missing information in the notes, the proportion of missing data has decreased since MBRRACE reporting began; however, if this information is not available, either because it was not recorded or women were not asked, this may not be possible.<br><br>There are a number of factors that can contribute to missing data and this can vary by

	variable. For instance, social risk factors such as domestic abuse and child abuse are often the most poorly recorded factors in notes. It can also vary by the triennia in which the data were collected as MBRRACE have improved the surveillance and data checking over the course of the programme. Women who were unbooked (i.e. died early in pregnancy prior to being known to maternity services or had a concealed pregnancy) will often have large amounts of missing data as this will not have been captured at the initial antenatal appointment. For the cohort studied in this paper, there does not appear to be a clear bias towards missing data for women who did or did not have CSC involvement.<br><br>To address missingness on socio-demographic, medical and health-related variables, we have included two supplementary tables (Table S2 and 3), with reference to these tables in the main manuscript.
Although some limitations of the case note review and given, there needs to be a more thorough exploration of the limitations of the study within the discussion (including the surveillance data element).	Many thanks for this suggestion.<br><br>We have explored limitations in more detail in a designated section of the manuscript. We hope you will find this section has now been strengthened and sufficiently explored the most pertinent limitations of the study.
Abstract results – please define what age range is meant by "younger", and what the comparator is.	Thank-you, we have included this now in the abstract results.
The methods of statistical analysis used to derive the relative risks should be stated in the design section, and RR should be given in full the first time it is used.	We have defined RR in full the first time this abbreviation is used (Rate Ratio).<br><br>We have not derived a relative risk, but a rate ratio, to compare the incident rates of CSC involvement for different groups (age, ethnicity, IMD quintile) amongst all women who died. To compare incident rates, the 'iri' command was used and this has been expanded on in the methods section.
Similarly, the comparator group for ethnicity (white?) and deprivation (is this all other groups, or least deprived?) needs to be stated.	Thank-you, we have included this now in the abstract results.
Please give the percentage of women who were excluded because of a lack of information on the surveillance form relating to CSC involvement.	We have reported the number of women with this information missing (n=244, 14%), which is

How representative were those who were excluded for this reason?	in line with previous MBRRACE-UK reports and their approach to missingness.<br><br>We believe further analysis of how representative this group is would be beyond the scope of this project, and would not be beneficial. Often women with missing information in one section of the surveillance form will have missing information in most sections. For instance, among women where it is unknown if they had CSC involvement, information is missing for employment (50%), living arrangements (58%), domestic abuse (90%), ethnicity (50%), pre-existing medical conditions (58%) and mental health problems (66%).
Is there a justification for grouping ethnicity into the 5 broad categories? If so, this should be given.	Five broad categories were used due to small numbers in some of the categories for women with social care involvement. This has been added to the methods as follows:<br><br>*Socio-demographic covariates to identify different population groups included maternal age, social deprivation and ethnicity. Social deprivation was derived from quintiles of the Index of Multiple Deprivation (IMD). Ethnicity data were grouped as five aggregated categories used in the UK Census classification (Asian, Black, Mixed or multiple ethnicity, Other or White), due to small numbers in some of the categories for women with CSC involvement.*<br><br>We hold more granular ethnicity data, as each woman's data are input in line with the 18 categories within the UK census coding. However, we needed to suppress numbers smaller than 5 as this is personal information. Even when aggregating the data in 8 ethnicity categories, there were still some with only 2-3 women.
Rather than stating that you used the 'iri' command, please describe how this command calculates the mortality rate ratio.	Thank you for this suggestion. The rate ratio presented is not a mortality rate, but an incidence rate amongst women who died, and this has been amended in the methods. We have made the following change in the manuscript to describe the use of 'iri':<br><br>*Using the incidence-rate ratio calculator command in Stata ('iri'), the incident rates by CSC involvement for different population groups*

	(age, IMD quintile and ethnicity) amongst women who died were calculated.
Table 2 – how the "rate" column has been labelled is a little confusing. Rather than a rate, I think this is just the percentage of women who died, who had CSC involvement. Suggest this is relabelled.	Thank you for this observation. You are correct that this is also a proportion as the rate is reported per 100 deaths. In this instance we believe reporting the numbers in either fashion would be appropriate as the relative risk for proportion would be the same as the rate ratio for incidence rate. We have opted to keep this as incidence rate to maintain consistency with other MBRRACE reporting.
Table 3 – above substance use and below BMI, there is a row title missing. Received any antenatal care – row titles missing.	Apologies, it seems some issues regarding table formatting occurred when uploading them on the portal. The tables have now been embedded in the main manuscript and this should have resolved the issue.
Figure 2 – typo "naternal"	We have corrected this typo
Supplementary table S2 is interesting. Do you have data on the non-sampled cases that can readily be extracted, so that we can see how representative the sample is?	The demographic variables are available for the entire cohort through the surveillance data, as presented in table 1 in the paper, which are largely similar to those in S2. However, most of the variables in S2 under "social complexity and social care involvement" are not available in the surveillance data but were manually recorded from the health records when competing the case note review. These therefore could not be directly compared.

VERSION 2 – REVIEW

**REVIEWER NAME**	Harron, Katie
**REVIEWER AFFILIATION**	
**REVIEWER CONFLICT OF INTEREST**	None
**AI DISCLOSURE**	No
**DATE REVIEW RETURNED**	21-May-2025

**GENERAL COMMENTS**	The authors have addressed my previous comments and the paper is much clearer now. I have no further comments.
